# Primary Erythrophagocytic Low-Grade Splenic T-Cell Lymphoma in a Cat: Clinical Case and Literature Review

**DOI:** 10.3390/ani16203234

**Published:** 2026-10-14

**Authors:** Aurora Barbetta, Valentina Cremonini, Maria Teresa Antognoni, Francesco Birettoni, Ilaria Porcellato, Carlo Masserdotti, Claudio Lazzari, Arianna Miglio

**Affiliations:** 1 Department of Veterinary Medicine, University of Perugia, 06124 Perugia, Italy; aurora.barbetta@dottorandi.unipg.it (A.B.); cremonini.valentina2@gmail.com (V.C.); francesco.birettoni@unipg.it (F.B.); ilariaporcellatodvm@gmail.com (I.P.); 2 Idexx Laboratories Italia S.r.l., 20149 Milano, Italy; carlo-masserdotti@idexx.com (C.M.); claudio-lazzari@idexx.com (C.L.)

**Keywords:** feline, lymphoma, spleen, erythrophagocytosis, T-cell lymphoma, PARR, xenocannibalism

## Abstract

Lymphoma is a common hematopoietic neoplasm in cats; yet hepatosplenic forms remain rare. This report describes a unique case of low-grade erythrophagocytic splenic T-cell lymphoma with suspected peripheral blood involvement in a six-year-old neutered male domestic shorthair cat. The patient presented with severe non-regenerative macrocytic normochromic anemia and moderate splenomegaly. Blood smears revealed mononucleated cells exhibiting erythrophagocytosis. Splenic cytology and flow cytometry were consistent with a reactive process, leading to a presumptive diagnosis of immune-mediated anemia. The condition proved refractory to immunosuppressive therapy with prednisolone and cyclosporine. After splenectomy, histopathology showed diffuse erythrophagocytosis and white pulp hyperplasia. Definitive diagnosis was achieved only by PARR analysis, which demonstrated a monoclonal T-cell receptor gene rearrangement within a polyclonal background. This confirmed a neoplastic T-cell population despite the mature morphology of the lymphocytes. Treatment with chlorambucil and prednisolone resulted in progressive resolution of the anemia. The cat remains alive 13 months after splenectomy. This case highlights the diagnostic challenges of low-grade lymphomas, in which reactive features can mask neoplasia, and underscores the critical role of PARR analysis in differentiating monoclonal T-cell proliferation from complex reactive processes in feline splenic disorders.

## Simple Summary

Lymphoma is a common neoplasm in cats, but the “erythrophagocytic” splenic form is exceptionally rare and diagnostically challenging. A six-year-old cat presented with severe, non-regenerative, macrocytic anemia. Initial diagnostics (including cytology and flow cytometry) suggested immune-mediated hemolytic anemia, as the lymphoid cells appeared mature. However, erythrophagocytosis was observed and the patient failed to respond to immunosuppressive therapy. Splenectomy was performed and histopathology revealed marked erythrophagocytosis. Definitive diagnosis required Polymerase chain reaction amplification for Antigen Receptor Rearrangements (PARR), which identified a monoclonal T-cell population. Treatment with chlorambucil led to complete resolution of the anemia. This case demonstrates that PARR is often essential for distinguishing low-grade neoplastic processes from reactive lymphoid hyperplasia in feline splenic disorders.

## 1. Introduction

Lymphoma is the most common hematopoietic neoplasm in cats. It typically arises from lymphoid tissues and often presents in a multicentric (nodal) form; however, extranodal forms originating from other tissues and organs are also well recognized [1,2].

The most frequent extranodal forms in cats include intestinal [3], mediastinal [4], nasal [5] and renal [6] lymphomas, with additional reports involving the upper respiratory tract [7], lungs [8], central nervous system, spinal cord [9], eyes [10], heart [11], skin [12] and other tissues [1,2]. Documented cases of feline primary hepatic [13,14,15,16] and hepatosplenic lymphomas [17,18,19] remain rare.

Hepatosplenic T-cell lymphoma (HS-TCL) is an exceptionally rare subtype of extranodal T-cell lymphoma in cats, with only a limited number of cases described in the veterinary literature [17,18,19]. The previously reported feline cases were characterized by the concurrent involvement of both the spleen and liver, typically with sinusoidal infiltration by neoplastic T cells [17] and, in some cases, erythrophagocytosis [17,18,19]. Notably, none of the previously described cases showed an exclusive splenic localization, as hepatic involvement was consistently documented alongside splenic infiltration.

Accurate diagnosis, grading [20] and immunophenotyping [21] are essential for treatment selection and prognosis [4]. The diagnostic process for suspected lymphoproliferative disorders usually includes cytological and histological examination, supplemented by immunophenotyping via flow cytometry and/or immunohistochemistry [22].

High-grade lymphomas are characterized by medium-to-large lymphoid blasts with a high mitotic index [21,23]. They are readily identified by cytology and histology and are associated with rapid progression and poor prognosis [21]. In contrast, low-grade lymphomas are more challenging to diagnose because the neoplastic cells closely resemble physiological small lymphocytes and are often accompanied by a mixed inflammatory infiltrate (plasma cells, granulocytes, and other inflammatory cells). These tumors typically produce milder clinical signs and carry a more favorable prognosis [24].

Feline lymphomas are predominantly of B- or T-cell origin. B-cell lymphomas are usually medium-to-high grade, whereas T-cell lymphomas may be high- or low-grade [21].

Polymerase chain reaction amplification for Antigen Receptor Rearrangement (PARR) is a valuable ancillary diagnostic tool that helps confirm clonality and lineage in ambiguous low-grade cases [25].

Here, to the authors’ knowledge, we describe the first reported case of feline erythrophagocytic low-grade T-cell lymphoma confined to the spleen and associated with suspected peripheral blood involvement. This distinctive presentation, characterized by isolated splenic disease without hepatic involvement and the presence of circulating neoplastic cells, expands the currently described clinical spectrum of this rare entity. Furthermore, this case highlights the pivotal role of PARR in establishing the diagnosis when routine diagnostic procedures are inconclusive.

## 2. Case Description

A six-year-old neutered male domestic shorthair cat was referred to the Veterinary Teaching Hospital of the University of Perugia (PG-VTH) with a 1.5-month history of severe non-regenerative macrocytic normochromic anemia, thrombocytopenia, hypoproteinemia, hyperglobulinemia, hyperbilirubinemia, progressive weight loss and inappetence. The cat had indoor and outdoor access and received neither vaccinations nor parasite prevention.

The referring veterinarian had initiated empirical therapy with doxycycline (10 mg/kg q24h), prednisolone (2.5 mg/kg q24h), hepatoprotectants, B-complex vitamins and transdermal mirtazapine. Because of persistent clinical signs and severe anemia after 10 days, the cat was referred for further evaluation.

At PG-VTH, the cat underwent physical examination, blood sampling for complete blood count (Idexx ProCyte Dx^®^ Hematology Analyzer, IDEXX Laboratories, Inc., Westbrook, ME, USA) and blood smear evaluation, serum biochemical analysis (Hitachi-904 Boehringer, Mannheim, Germany), serum protein electrophoresis (IDEXX Laboratories, Inc.), rapid test for the most common feline infectious diseases (Dirofilaria immitis, FIV and FeLV, SNAP^®^ Feline Triple^®^ Test, IDEXX Laboratories, Inc.), real-time PCR assay for *Mycoplasma* spp. (IDEXX Laboratories, Inc.), total thyroxine concentration (T4) (Catalyst One^®^ Chemistry Analyzer, IDEXX Laboratories, Inc.), coagulation tests (prothrombin time-PT, partial thromboplastin time-PTT, Coag Dx Analyzer^®^, IDEXX Laboratories, Inc.), an abdominal ultrasound and splenic, liver and bone marrow sampling for cytology.

At physical examination, the cat was normothermic and alert, but had pale and sub-icteric mucous membranes.

**Figure 1:**
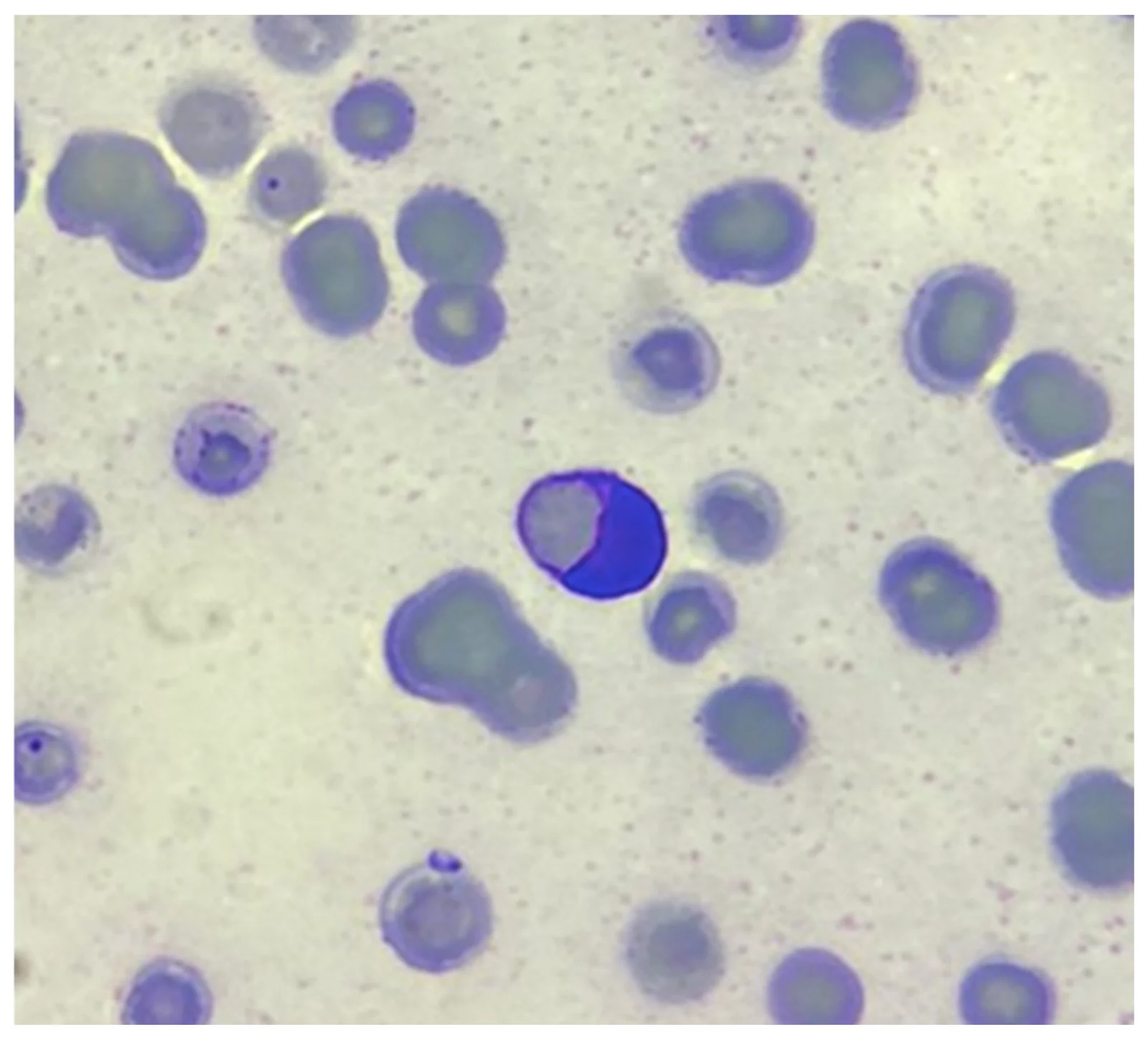
May–Grünwald–Giemsa-stained peripheral blood smear showing round cells exhibiting erythrophagocytosis. Original magnification 1000×.

A CBC showed hyporegenerative macrocytic normochromic anemia. Platelet counts were within the reference interval and were confirmed by blood smear examination to exclude the potential presence of platelet clumping (Table 1). Blood smear examination revealed moderate anisocytosis, mild polychromasia, several Howell-Jolly bodies and occasional nucleated red blood cells (NRBC). Moreover, a small number of small to medium-sized, mononucleated cells showing erythrophagocytosis were observed (Figure 1).

Biochemical analysis showed mild hyperbilirubinemia (total bilirubin (TBIL) 1.44, reference range (RR) 0.1–0.3 mg/dL; direct bilirubin (DBIL) 0.7, RR 0.0–0.3 mg/dL; indirect bilirubin (IBIL) 0.74, RR 0.0–0.3 mg/dL) and mild increases in serum transaminase activities (aspartate transaminase (AST) 103, RR 14–49 IU/L; alanine transaminase (ALT) 263, RR 6–80 UI/L).

T4 concentration was within RR (3.0 µg/dL, RR: 0.8–4.7 µg/dL); PT and PTT were normal [(PT 16.0 s, RR 15.0–22.0 s) and (PTT 76 s, RR 65.0–119.0 s)]. Serum protein electrophoresis was unremarkable, and serology and PCR for *Mycoplasma* spp. were negative, as were the rapid tests for *Dirofilaria immitis*, FIV and FeLV.

Abdominal ultrasonography demonstrated moderate splenomegaly (1.45 cm thickness) with normal echogenicity and echostructure, together with nephromegaly (5 × 3 cm) and cortical thickening (1 cm). No other relevant abdominal abnormalities were detected.

**Figure 2:**
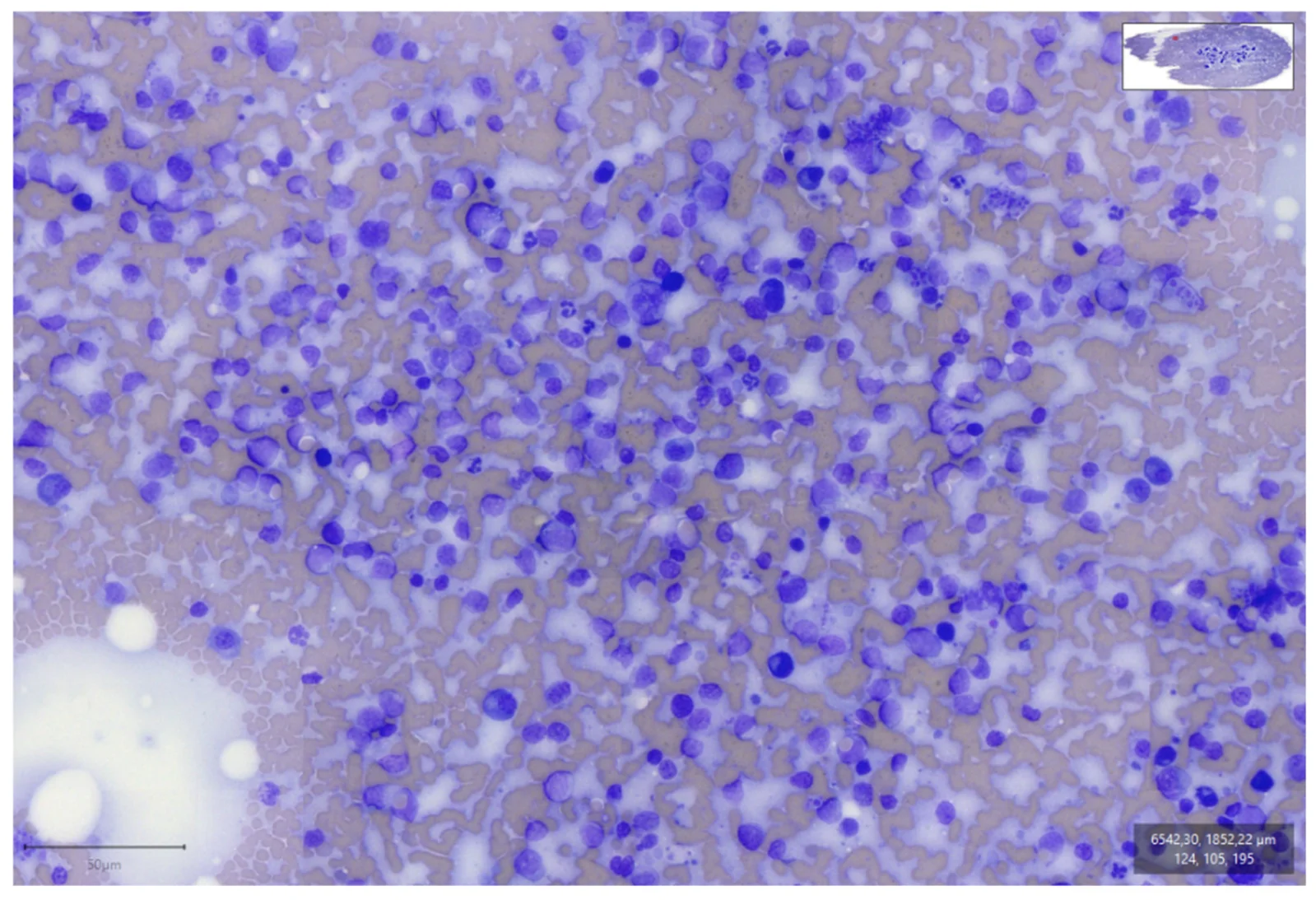
May-Grünwald–Giemsa-stained cytological smear from a fine-needle aspirate of spleen showing polymorphic lymphoid population, round cells exhibiting signs of erythrophagocytosis and extramedullary erythropoiesis. Original magnification, 200×.

**Figure 3:**
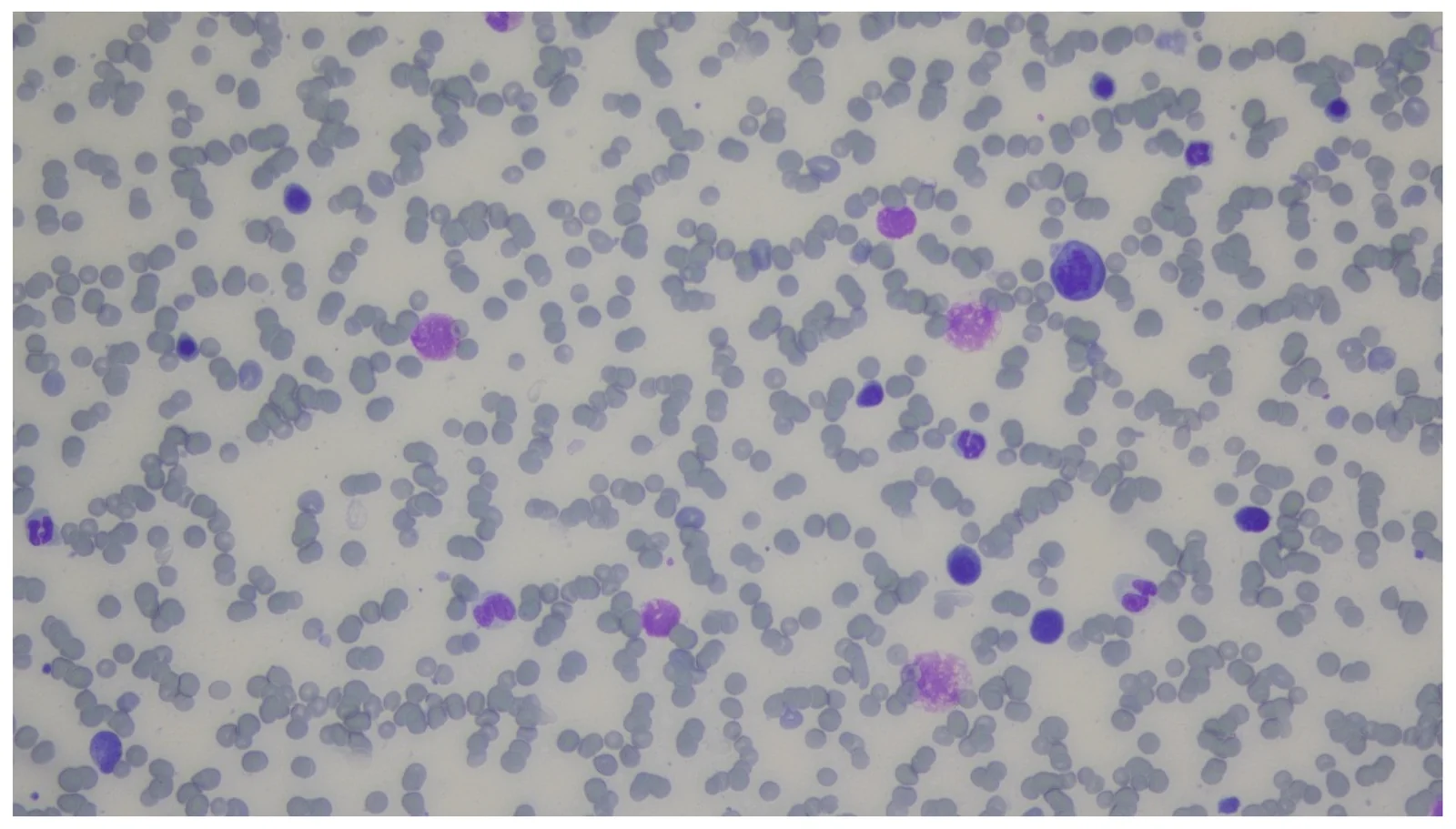
May-Grünwald–Giemsa-stained cytological smear from a fine-needle aspirate of bone marrow, paucicellular sample, erythroid precursors and a mitosis. Original magnification 400×.

Splenic cytology showed a polymorphic lymphoid population (small 35%, medium 35% and large 10% lymphocytes, histiocytoid cells 15% and plasma cells 5%) accompanied by extramedullary erythropoiesis. Interestingly, about 15% of the round cells exhibited signs of erythrophagocytosis. Therefore, cytological examination revealed a condition of lymphoid and plasma cell hyperplasia/dysplasia associated with erythrophagocytosis and extramedullary hematopoiesis (Figure 2). Based on the morphological features of the cells, which showed features overlapping those of both lymphoid and histiocytic/macrophage cell lineages, together with the prominent erythrophagocytic activity, the differential diagnoses included a round cell neoplasm or a histiocytic/macrophage disorder, such as hemophagocytic syndrome. Bone marrow cytology showed a predominant erythroid lineage with preservation of the maturation pyramid (total erythroid lineage: 63%; proerythroblasts: 1%; basophilic erythroblasts: 5%; polychromatic erythroblasts: 20%; acidophilic erythroblasts: 37%). The myeloid lineage was well represented at all stages of maturation, with a predominance of mature neutrophils and no morphological abnormalities were observed (total myeloid lineage: 31%; myeloblasts/promyelocytes: 1%; myelocytes: 2%; metamyelocytes: 3%; neutrophils: 25%; eosinophils: 0%). The myeloid-to-erythroid ratio was 0.5 (RR: 1.2–2.2). Lymphocytes represented approximately 6%. Based on these results, the interpretation of bone marrow cytology was erythroid hyperplasia with no morphological abnormalities (Figure 3) as a regenerative response to peripheral anemia.

Given the severity of anemia, the cat was hospitalized and received a blood transfusion. A clinical improvement was achieved, and due to the owner’s decision to avoid further diagnostic tests, the cat was discharged with prednisolone (1 mg/kg q12h) plus cyclosporine (5 mg/kg q12h) under a presumptive diagnosis of immune-mediated hemolytic anemia.

One month later, the cat was re-evaluated for anorexia. During the clinical examination, it was alert and normothermic, but the icterus appeared pronounced. CBC and biochemistry analysis worsened and showed a severe poorly regenerative macrocytic anemia (Table 1) and a mild increase in concentrations of hepatic enzymes and bilirubin (TBIL 3.71, RR 0.1–0.3 mg/dL; DBIL 2.16, RR 0.0–0.3 mg/dL; IBIL 1.55, RR 0.0–0.3 mg/dL, AST 114, RR 14–49 IU/L; ALT 192, RR 6–80 UI/L). The cat was readmitted to PG-VTH for an additional blood transfusion and, to achieve a definitive diagnosis, additional diagnostic procedures were performed with the owner’s consent. Splenectomy and liver biopsy for spleen and liver histologic examination and immunohistochemistry were performed. Moreover, fine-needle aspiration biopsy (FNAB) of splenic tissue and peripheral blood were submitted for flow cytometry (Laboratory of the Department of Veterinary Sciences, University of Turin, 10095 Turin, Italy).

The clinical condition and the laboratory results (Table 2) of the cat improved soon after the splenectomy and the animal was discharged with immunosuppressive (prednisolone and cyclosporine) and antibiotic therapy (amoxicillin and clavulanic acid) pending the results of the aforementioned diagnostic tests.

Macroscopic examination of the spleen showed no detectable lesions, except a mild diffuse enlargement of the organ. Histology revealed marked congestion and follicular hyperplasia of the white pulp, with occasional prominent germinal centers exhibiting increased mitotic activity. The remaining splenic pulp was characterized by a diffuse infiltration of round cells often engaged in erythrophagocytosis. These cells were characterized by a moderate to abundant finely granular cytoplasm often containing 1 or more red blood cells. The nucleus was rounded, with finely granular chromatin and an occasionally visible single nucleolus. Anisocytosis and anisokaryosis were minimal and no mitoses were seen. Often, the nucleus was indented, compressed and displaced because of the presence of intracytoplasmic red blood cells. Based on their morphological features, the histological classification of these cells remained uncertain, as they could potentially be classified as either lymphocytes or macrophages. However, the presence of erythrophagocytic activity supported their interpretation as macrophages.

**Figure 4:**
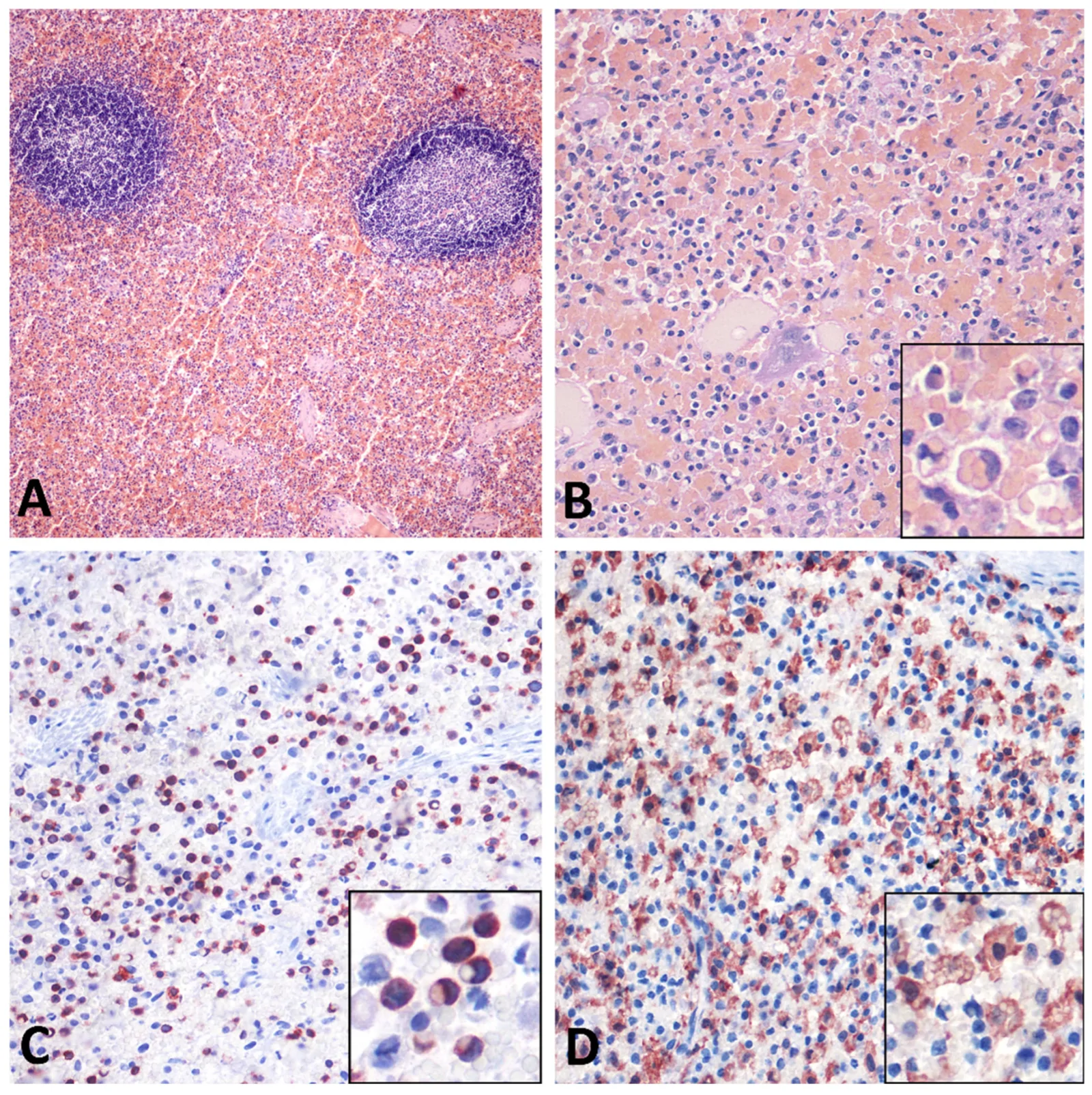
(**A**) Low magnification of spleen sections showed disseminated follicular-like structures with mild hyperplasia. The splenic red pulp is hyperemic. (Hematoxylin and eosin, 10×). (**B**) Within the red pulp, numerous cells showed erythrophagocytosis (inset). The number of phagocytosed red blood cells was variable (1–5). (Hematoxylin and eosin, 40×). (**C**) Most of the cells phagocytosing a single erythrocyte were CD3-positive, characterized by an indentation of the nucleus, which was displaced by intracytoplasmic red blood cells (inset). CD3+ lymphocytes were disseminated and not forming aggregates or nodules in the spleen. (AEC and hematoxylin, 40×). (**D**) IBA1+ histiocytes were disseminated. Numerous IBA1-immunolabeled elements showed numerous intracytoplasmic erythrocytes (inset). (AEC and hematoxylin, 40×).

This cellular population was admixed with a significant number of small lymphocytes and mature plasma cells without atypia. Foci of extramedullary erythropoiesis and haemosiderin-laden macrophages were multifocally present. Histological diagnosis was hyperplasia of the white pulp with diffuse erythrophagocytosis and multifocal extramedullary hematopoiesis (Figure 4A,B). The possibility of a malignant hematological disorder presenting as hemophagocytic syndrome could not be ruled out.

Immunohistochemical examination of the spleen demonstrated that hemophagocytic cells were characterized by CD3 or IBA1 immunolabeling. Most CD3+ cells showing erythrophagocytosis were small and contained only one erythrocyte (Figure 4C), whereas IBA1+ cells usually contained more red blood cells (Figure 4D), which were larger in size and morphologically similar to the other splenic histiocytes. CD20 immunolabeling was limited to germinal centers of lymphoid follicles and scattered B cells in splenic parenchyma, demonstrating lymphoid splenic polymorphism.

Histological examination of the liver revealed moderate hepatopathy characterized by cholestasis, moderate hydropic degeneration of hepatocytes and mild periportal infiltration of mature lymphocytes.

Flow cytometry of the splenic pulp, which showed a certain degree of necrosis, revealed a mixed population of small lymphocytes of variable phenotype (MHCII+CD5+CD21-; MHCII+CD5-CD21+; MHCII+CD5-CD21-; CD4+CD8-; CD4-CD8+) and a smaller component of large lymphoid cells (CD5-CD21+; CD5+CD21-) and neutrophils (CD18+CD11b+). The interpretation of the splenic flow cytometry indicated a hyperplastic/reactive lymphoid population.

Flow cytometry of peripheral blood revealed a predominance of CD11B+ cells (neutrophils and monocytes) and a smaller component consisting of small lymphoid cells (CD5+CD21-; CD5-CD21+; CD4+CD8-; CD4-CD8+). Flow cytometry of peripheral blood revealed a normal leukocyte population without aberrant scatter or immunophenotype.

Approximately two months after splenectomy, the hematological values again deteriorated (Table 2). To exclude a lymphoid neoplastic origin of the disease, PARR was performed on unstained sections from formalin-fixed, paraffin-embedded splenic tissue. Due to financial constraints, clonality assessment was limited to the splenic sample, which was selected because histological examination revealed a substantial population of morphologically suspicious cells. In contrast, erythrophagocytic mononuclear cells were only rarely observed in peripheral blood. DNA was automatically extracted using the Maxwell^®^ RSC 48 Instrument (Promega, Madison, WI, USA) and the Maxwell^®^ RSC DNA FFPE Kit (Promega, Madison, WI, USA), according to the manufacturer’s instructions. The clonality assay was performed at a commercial laboratory (Genefast, Forlì, Italy) according to previously published methods [26,27,28], using three PCRs targeting the T-cell receptor (TCR) gene rearrangements and four PCRs targeting the B-cell receptor (BCR) gene rearrangements, together with an additional panel targeting the immunoglobulin lambda light chain (IgL) and kappa deleting element (Kde). BCR gene rearrangements were assessed by targeting the immunoglobulin heavy chain (IGH), IgL, and Kde loci, whereas T-cell clonality was assessed by targeting the T-cell receptor gamma (TRG) locus. Positive and negative controls were included and produced the expected results.

PCR products underwent GeneScanning analysis on an automated SeqStudio Genetic Analyzer (Thermo Fisher Scientific, Waltham, MA, USA). GeneScanning electrophoretic patterns were interpreted according to Keller et al. (2016) [26], integrated with the laboratory’s internal method. Samples showing one or two sharp and reproducible peaks, or multiple distinct peaks for multi-cassette loci such as TCR, were classified as clonal. Samples exhibiting three to five reproducible peaks were classified as oligoclonal, whereas samples presenting a normally distributed (Gaussian) curve were classified as polyclonal. The emergence of clonal peaks was interpreted as most indicative of clonal selection, whereas oligoclonal and polyclonal results were considered consistent with a reactive population. Samples were analyzed in duplicate to reduce the possibility of pseudoclonality.

**Figure 5:**
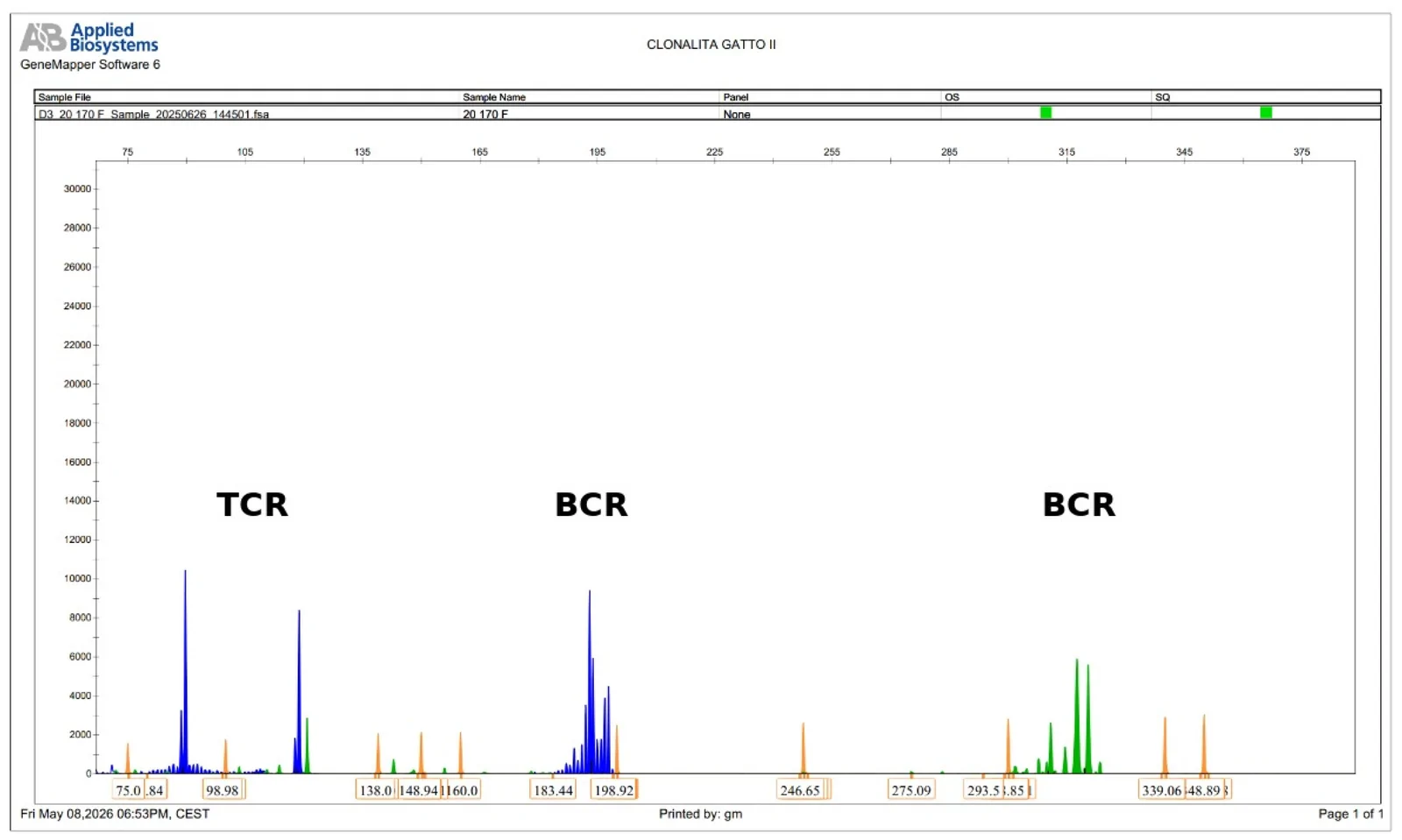
GeneScan electropherograms from PARR analysis performed on unstained sections of formalin-fixed, paraffin-embedded splenic tissue. The T-cell receptor (TCR) analysis shows a prominent, reproducible clonal peak pattern, consistent with a monoclonal T-cell population within a polyclonal background. The B-cell receptor (BCR) analysis shows a broad, Gaussian-like distribution of peaks, consistent with a polyclonal B-cell population.

PARR demonstrated a polyclonal B-cell receptor gene (BCR) rearrangement and a monoclonal T-cell receptor gene (TCR) rearrangement against a polyclonal background (Figure 5). These findings indicated a clonal lymphoid proliferation suggestive of T-cell lymphoma. Based on this result, the definitive diagnosis of low-grade erythrophagocytic splenic T-cell lymphoma was made.

Based on this result, the definitive diagnosis of low-grade erythrophagocytic splenic T-cell lymphoma was made and cyclosporine was replaced with an antineoplastic drug (chlorambucil, 2 mg/cat orally every 48 h), combined with prednisolone 2 mg/kg q24h. Over the subsequent months, the anemia progressively resolved (Table 2), and the cat was alive at the time of writing (13 months after splenectomy and initiation of chemotherapy).

## 3. Discussion

To the best of our knowledge, this is the first reported case of low-grade erythrophagocytic splenic T-cell lymphoma in a cat. In the veterinary literature, only one previous low-grade T-cell lymphoma involving both spleen and liver has been described [17], together with two high-grade erythrophagocytic hepatosplenic T-cell lymphomas [18,19]. Erythrophagocytic lymphomas remain rare in animals; a single canine hepatic case has been reported in addition to the feline cases [29]. In human medicine, however, hepatosplenic Tγδ lymphoma could be associated with erythrophagocytosis [30].

In the present case, anamnestic data, clinical evaluation, diagnostic imaging investigations and laboratory findings were nonspecific and included severe hyporegenerative anemia, splenomegaly, and mild hepatopathy. Similar features were described by Carter et al. (2008), although hepatomegaly was also evident in that report [17].

Blood smear, bone marrow, and spleen cytology were suggestive of immune-mediated hemolytic anemia (IMHA) as the primary differential diagnosis. This suspicion was supported by the presence of hyporegenerative macrocytic anemia, erythroid hyperplasia in the bone marrow, and peripheral mononucleated erythrophagocytic round cells. Correspondingly, splenic evaluation revealed lymphoid hyperplasia/dysplasia, extramedullary erythropoiesis, and mononucleated erythrophagocytic cells morphologically identical to those observed in peripheral blood. These findings were similar to those reported by Carter et al. (2008), who described round cells exhibiting erythrophagocytosis in the spleen and liver, but not in the peripheral blood [17]. In contrast, in the present case, morphologically similar erythrophagocytic cells were observed both in the spleen and peripheral blood, while bone marrow cytology excluded neoplastic infiltration. This finding further supports the distinctive presentation of our case, characterized by isolated splenic involvement with suspected peripheral blood involvement.

Accordingly, the cat was treated with adjunctive immunosuppressive therapy and received a blood transfusion [31]. Unfortunately, a Coombs test could not be performed, as immunosuppressive corticosteroid therapy had been initiated 10 days prior to our initial evaluation, which could have rendered the results unreliable [32].

The lack of response to intensified immunosuppression raised suspicion of underlying neoplasia and prompted splenectomy. Splenectomy may also be recommended as adjunctive therapy in cases of IMHA in humans and dogs [31,33], although the efficacy remains uncertain in cats [34].

Histological examination of the spleen was inconclusive, showing white pulp hyperplasia with diffuse lymphocyte distribution and erythrophagocytosis by histiocytic/macrophage-like cells. Based on these findings, a malignant hematopoietic disorder with associated hemophagocytic syndrome cannot be ruled out [34].

Interestingly, splenic immunohistochemistry confirmed the T-lymphocyte origin (CD3+) of the erythrophagocytic cells, distinguishing them from macrophages (IBA1+), which were also involved, albeit to a lesser extent, in erythrophagocytosis and physiological erythrocatheresis. Immunohistochemistry also highlighted lymphoid polymorphism, with B lymphocytes (CD20+) admixed with the T-cell population.

Our findings partially differ from those reported by Carter et al. (2008), who demonstrated the presence of CD3+ erythrophagocytic cells in both the spleen and liver; in their case, the marked involvement of both organs facilitated the definitive diagnosis of lymphoma [17]. In the present case, the less pronounced T-cell infiltration of the spleen, together with the absence of neoplastic involvement of the liver, made the diagnosis more challenging. Given the pleomorphic histological appearance, flow cytometry was performed to further characterize the splenic cell phenotype. However, this technique only confirmed the presence of a polymorphic leukocyte population typical of reactive or hyperplastic conditions [22]. In neoplasms characterized by disseminated infiltration rather than nodular or mass-forming proliferations, the sensitivity of flow cytometry is limited [35].

Since none of the above investigations resolved the uncertainty between a hyperplastic/reactive lymphoid process secondary to an immune-mediated disorder and a malignant hematological neoplasm, PARR was performed to better define the lymphoid cell population. Notably, PARR was the only diagnostic tool that identified a monoclonal proliferation of T-cell lineage admixed with polyclonal background, thereby establishing the definitive diagnosis of low-grade T-cell lymphoma, likely at an early stage given the incomplete parenchymal involvement and the presence of a mixed inflammatory infiltrate [25].

PARR is a molecular assay that evaluates the mono- or polyclonality of B- and T-lymphocyte antigen receptors. These receptors are encoded by genes with variable nucleotide sequences; each lymphocyte undergoes gene rearrangement to maximize antigen receptor specificity. Rearrangement of the immunoglobulin heavy chain (IGH) variable region indicates B-cell lineage, whereas rearrangement of the T-cell receptor gamma (TCRG) gene indicates T-cell lineage. Neoplastic lymphoid cells originate from a single clone and maintain the same antigen receptor rearrangement (IGH or TCRG). PARR employs PCR to amplify the gene segments encoding these antigen receptors [25,26]. In the present case, PARR was fundamental for achieving a definitive diagnosis due to its ability to detect a clonal population within a predominantly reactive background [22].

High-grade lymphomas are typically characterized by neoplastic cells that are readily identifiable on cytology and histology because of their distinct morphological features, such as medium-to-large size, characteristic chromatin pattern, and prominent nucleoli [18,19,21]. In contrast, feline low-grade lymphomas often consist of neoplastic cells resembling small lymphocytes normally found in lymphoid tissues, surrounded by a heterogeneous reactive inflammatory infiltrate [27,36]. In such cases, PARR analysis may be recommended to avoid misdiagnosis.

Although PARR is valuable for detecting neoplastic populations by identifying monoclonal peaks, it should not be used as a standalone diagnostic tool. Rather, it should be interpreted in conjunction with histology, immunohistochemistry, and flow cytometry to complete the phenotypic characterization of suspected neoplastic lymphoid cells [37]. In the present case, immunohistochemistry demonstrated that the erythrophagocytic cells expressed CD3, supporting their T-cell lineage, while PARR confirmed the presence of a monoclonal T-cell population within the splenic lesion. Although PARR, performed on the tissue sample as a whole, did not allow for the direct demonstration that the detected monoclonal population specifically corresponded to the CD3+ erythrophagocytic cells, the combined morphological, immunohistochemical, and molecular findings strongly support the interpretation that these cells were part of the clonal T-cell population.

Confirmation of the neoplastic nature of the disease was crucial for the patient, as splenectomy alone was insufficient to control the condition. This diagnosis allowed the initiation of antineoplastic chemotherapy, which enabled the cat to survive for a considerable period.

Erythrophagocytosis is a pathological phenomenon characterized by the phagocytosis of mature erythrocytes or their precursors by activated or transformed macrophages. Although physiologically restricted to the removal of senescent erythrocytes, it becomes clinically relevant when excessive and systemic, often secondary to diseases such as IMHA or hemophagocytic syndrome [32,38,39,40,41,42].

Recent literature has shown that neoplastic cells can acquire the ability to phagocytose other cells, a phenomenon termed “xenocannibalism” [43]. Specifically, “erythrocytic xenocannibalism” refers to the ingestion of red blood cells by cells that are not traditionally phagocytic. In cats and dogs, this phenomenon has been primarily observed in association with lymphoid and plasma cell neoplasms and mast cell tumors [43,44,45]. In dogs, it has also been described in other neoplasms, including sarcomas such as hemangiosarcomas and osteosarcomas [29].

Initial peripheral blood and splenic examinations revealed round erythrophagocytic cells of uncertain lineage, prompting a differential diagnosis between hemophagocytic syndrome and lymphoma with erythrocytic xenocannibalism. Immunohistochemistry of the spleen ultimately confirmed their T-lymphocyte lineage. Unfortunately, the erythrophagocytic cells observed in peripheral blood were not immunophenotyped, and their T-cell lineage, therefore, remains suspected but unconfirmed.

To our knowledge, this represents the first reported case of low-grade erythrophagocytic T-cell lymphoma with only splenic involvement in which erythrophagocytosis was evident in both peripheral blood and spleen. In the veterinary literature, erythrophagocytosis in both high- and low-grade T-cell lymphomas in cats has been described only in splenic, hepatic, and bone marrow tissues [17,18,19]. Peripheral blood erythrophagocytosis has previously been reported in only one case of chronic lymphocytic leukemia in a cat [44]. While the erythrophagocytic cells identified in peripheral blood were morphologically similar to the CD3+ erythrophagocytic cells observed in the spleen, their T-cell lineage could not be directly demonstrated. Therefore, peripheral blood involvement by the neoplastic T-cell population was suspected but could not be definitively confirmed.

Erythrophagocytic cells observed in lymphoid neoplasms (lymphomas or lymphocytic leukemias) in dogs and cats are typically of cytotoxic T-cell origin [17,18,19,29,44,46]. In this case, flow cytometry on splenic fine-needle aspirates revealed a population of CD4−/CD8+ lymphocytes, consistent with cytotoxic activity. This cell lineage has been reported to exhibit aberrant erythrophagocytic activity [18].

In human medicine, the majority of lymphoid erythrophagocytic neoplasms are of cytotoxic T-cell origin, both in lymphomas [47,48] and leukemias [49,50], although rare B-cell cases have been documented [51].

Primary splenic non-Hodgkin lymphomas are uncommon in humans and are mostly of B-cell lineage. T-cell splenic lymphomas are predominantly hepatosplenic lymphomas [52], with only a few reported cases of primary splenic T-cell lymphomas [53,54]. These are diagnosed through a combination of histology, immunohistochemistry, and PARR, which allows confirmation of the T-cell immunophenotype [53,54,55]. Even in humans, achieving a definitive diagnosis is challenging due to nonspecific clinical signs and inconclusive morphological and immunophenotypic findings, particularly in early stages. Consequently, this neoplasm is considered underdiagnosed [53].

Unlike the present case and those reported in the veterinary literature, erythrophagocytosis in humans is primarily performed by histiocytic-macrophage cells, and the neoplastic lymphoid cells are typically CD4+. Moreover, splenectomy alone was often sufficient for complete resolution, without the need for adjunctive chemotherapy [53,56].

As observed in dogs, hepatosplenic lymphomas in humans are highly aggressive, with a poor prognosis and limited response to treatment. They infiltrate the spleen and liver but spare peripheral lymph nodes. These neoplasms arise from γδ-T lymphocytes, which possess phagocytic and cytotoxic capabilities, and erythrophagocytosis is observed in both neoplastic and histiocytic cells [55,56,57].

In the present case, the erythrophagocytic cells were neoplastic lymphocytes. Splenectomy alone was insufficient to control the disease, requiring chemotherapeutic intervention. Whether the present case represents an early form of hepatosplenic lymphoma in which hepatic infiltration was not yet detectable remains speculative.

Some limitations of this study include the inability to perform specific tests to definitively exclude IMHA (such as the Coombs test) and the lack of post-treatment cytological and PARR follow-up of blood, bone marrow, and liver owing to the owner’s decision.

## 4. Conclusions

This case describes a rare presentation of low-grade feline erythrophagocytic splenic T-cell lymphoma. This condition should be included in the differential diagnosis of non-regenerative/hyporegenerative macrocytic anemia, splenomegaly, and erythrocytic xenocannibalism performed by neoplastic lymphoid cells.

In this case, cytological, histological, and immunophenotypic examinations were suggestive of a reactive process. However, peripheral blood and splenic parenchyma revealed aberrant round cells exhibiting erythrophagocytosis, whose neoplastic nature was confirmed only by immunohistochemistry of the spleen.

To avoid underdiagnosis of this entity, PARR analysis proved to be the definitive diagnostic tool, as it was the only method capable of detecting a monoclonal T-cell receptor gene rearrangement within a complex polyclonal background. This technique is particularly useful for identifying neoplastic lymphoid populations with mature morphological features and low-grade characteristics. PARR has demonstrated value not only for diagnosis but also for guiding therapeutic decisions.

## Figures and Tables

**Table 1:** Hematological parameters of the cat on first admission at PG-VTH (Day 0), and then at second admission at PG-VTH (Day 30) and the day of surgery (Day 31).

	Day 0	Day 30	Day 31 *	Reference Range
RBC (×10^6^/µL)	**1.65**	**1.56**	**3.0**	6.54–12.20
Hb (g/dL)	**4.0**	**3.1**	**5.5**	9.8–16.2
Hct (%)	**12.5**	**10.6**	**17.1**	30.3–52.3
MCV (fL)	**75.8**	**67.9**	**57.0**	35.9–53.1
MCH (pg)	**24.2**	**19.9**	**18.3**	11.8–17.3
MCHC (g/dL)	32	29.2	32.2	28.1–35.8
RDW (%)	**27.8**	**36.7**	**42.1**	15.0–27.0
Reticulocytes (%)	2	4.1	3.8	
Reticulocytes (×10^3^/µL)	36.2	**72.5**	**71.1**	3.0–50.0
WBC (×10^3^/µL)	8.19	8.36	6.64	2.87–17.02
Segmented neutrophils (×10^3^/µL)	6.18	5.98	5.09	2.30–10.29
Lymphocytes (×10^3^/µL)	1.51	1.80	1.19	0.92–6.88
Monocytes (×10^3^/µL)	0.51	0.43	0.33	0.05–0.67
Eosinophils (×10^3^/µL)	**0.05**	0.15	**0.03**	0.17–1.57
PLT (×10^3^/µL)	220	315	206	151–600
MPV (fL)	**23.6**	20.4	**23.4**	11.4–21.6
PCT (%)	0.27	0.24	0.48	0.17–0.86

RBC: red blood cells; Hb: hemoglobin; Hct: hematocrit; MCV: mean corpuscular volume; MCH: mean corpuscular hemoglobin; MCHC: mean corpuscular hemoglobin concentration; RDW: red blood cell distribution width; WBC: white blood cells; PLT: platelets; MPV: mean platelet volume; PCT: plateletcrit. Values outside the reference interval are shown in bold. Post-transfusion values are indicated by *.

**Table 2:** Hematological parameters of the cat 10 days after surgery (Day 10), and then at 60 days (Day 60) and after another 2 months (Day 120) and after another 5 months (Day 270).

	Day 10	Day 60	Day 120	Day 270	Reference Range
RBC (×10^6^/µL)	**3.53**	**2.56**	**3.41**	**5.59**	6.54–12.20
Hb (g/dL)	**6.1**	**4.7**	**6.4**	9.8	9.8–16.2
Hct (%)	**21.4**	**19.2**	**23.2**	**29.8**	30.3–52.3
MCV (fL)	**60.5**	**74.9**	**67.9**	**53.3**	35.9–53.1
MCH (pg)	17.2	**18.2**	**18.8**	17.6	11.8–17.3
MCHC (g/dL)	28.4	**24.3**	**27.7**	33.0	28.1–35.8
RDW (%)	22.3	**11.0**	15.2	20.2	15.0–27.0
Reticulocytes (%)	0.2	0.3	0.7	0.1	
Reticulocytes (×10^3^/µL)	5.8	7.0	22.9	8.3	3.0–50.0
WBC (×10^3^/µL)	**21.52**	7.72	8.82	7.14	2.87–17.02
Segmented neutrophils (×10^3^/µL)	**11.82**	4.39	5.26	6.27	2.30–10.29
Lymphocytes (×10^3^/µL)	**7.23**	2.51	2.77	**0.61**	0.92–6.88
Monocytes (×10^3^/µL)	**1.86**	**0.73**	0.63	0.23	0.05–0.67
Eosinophils (×10^3^/µL)	0.54	**0.09**	**0.14**	**0.03**	0.17–1.57
PLT (×10^3^/µL)	269	266	352	**142**	148–600
MPV (fL)	**24.4**	**26.6**	**24.0**	**22.9**	11.4–21.6
PCT (%)	0.65	0.65	0.85	0.33	0.17–0.86

RBC: red blood cells; Hb: hemoglobin; Hct: hematocrit; MCV: mean corpuscular volume; MCH: mean corpuscular hemoglobin; MCHC: mean corpuscular hemoglobin concentration; RDW: red blood cell distribution width; WBC: white blood cells; PLT: platelets; MPV: mean platelet volume; PCT: plateletcrit. Values outside the reference interval are shown in bold.

## Data Availability

No new data were created or analyzed in this study. Data sharing is not applicable to this article.

## Abbreviations

The following abbreviations are used in this manuscript:

PARR	Polymerase chain reaction amplification for Antigen Receptor Rearrangements
PG-VTH	Veterinary Teaching Hospital of the University of Perugia
CBC	Complete blood count
PT	Prothrombin time
PTT	Partial thromboplastin time
FNAB	Fine Needle Aspiration Biopsy
BCR	B-cell receptor
TCR	T-cell receptor
RBC	Red blood cells
Hb	Hemoglobin
Hct	Hematocrit
MCV	Mean corpuscular volume
MCH	Mean corpuscular hemoglobin
MCHC	Mean corpuscular hemoglobin concentration
RDW	Red blood cell distribution width
WBC	White blood cells
PLT	Platelets
MPV	Mean platelet volume
PCT	Plateletcrit
TBIL	Total bilirubin
DBIL	Direct bilirubin
IBIL	Indirect bilirubin
TP	Total proteins
ALB	Albumin
AST	Aspartate transaminase
ALT	Alanine transaminase
ALP	Alkaline phosphatase
CPK	Creatine phosphokinase
LDH	Lactate dehydrogenase
CREA	Creatinine
GLU	Glucose
Ca	Calcium
PHOS	Phosphorus
IMHA	Immune-mediated hemolytic anemia
TCRG	T-cell receptor gamma
IGH	Immunoglobulin heavy chain
